# Microbiological pattern of arterial catheters in the intensive care unit

**DOI:** 10.1186/1471-2180-10-266

**Published:** 2010-10-19

**Authors:** Li Zhang, Kadaba S Sriprakash, David McMillan, John R Gowardman, Bharat Patel, Claire M Rickard

**Affiliations:** 1 Research Centre for Clinical and Community Practice Innovation, Griffith University, Brisbane QLD 4111, Australia; 2 Bacterial Pathogenesis Laboratory, Queensland Institute of Medical Research, Herston, QLD 4006, Australia; 3 Bacterial Pathogenesis Laboratory, Queensland Institute of Medical Research, Herston, QLD 4006, Australia Griffith Medical Research College, a joint program of Griffith University and the Queensland Institute of Medical Research, QIMR, Herston, QLD 4006, Australia; 4 Department of Intensive Care Medicine, Royal Brisbane Women's Hospital, Herston, QLD 4029, Australia Research Centre for Clinical and Community Practice Innovation, Griffith University, Brisbane QLD 4111, Australia; 5 School of Biomolecular and Physical Sciences, Griffith University, Brisbane, QLD 4111, Australia; 6 Research Centre for Clinical and Community Practice Innovation, Griffith University, Brisbane QLD 4111, Australia

## Abstract

**Background:**

Intravascular catheter related infection (CRI) is one of the most serious nosocomial infections. Diagnostic criteria include a positive culture from the catheter tip along with blood, yet in many patients with signs of infection, current culture techniques fail to identify pathogens on catheter segments. We hypothesised that a molecular examination of the bacterial community on short term arterial catheters (ACs) would improve our understanding of the variety of organisms that are present in this niche environment and would help develop new methods for the diagnosis of CRI.

**Results:**

The whole bacterial community presenting on all ACs was evaluated by molecular methods, i.e., a strategy of whole community DNA extraction, PCR amplification followed by cloning and 16S rDNA sequence analysis. Ten ACs were removed from patients suspected of CRI and 430 clones from 5 "colonised" and 5 "uncolonised" (semi-quantitative method) AC libraries were selected for sequencing and subsequent analysis. A total of 79 operational taxonomic units (OTUs) were identified at the level of 97% similarity belonging to six bacterial divisions. An average of 20 OTUs were present in each AC, irrespective of colonisation status. Conventional culture failed to reveal the majority of these bacteria.

**Conclusions:**

There was no significant difference in the bacterial diversity between the 'uncolonised' and 'colonised' ACs. This suggests that vascular devices cultured conventionally and reported as non infective may at times potentially be a significant source of sepsis in critically ill patients. Alternative methods may be required for the accurate diagnosis of CRI in critically ill patients.

## Background

Intravascular catheters (IVCs) occupy a very important place in the day-to-day provision of healthcare in hospitals. Nearly 300 million IVCs are used yearly in USA alone [1]. Along with their undoubted advantages IVCs are also associated with life-threatening infections [2]. Every year, approximately 3,500 Australians [3] are diagnosed with catheter-related bloodstream infections and up to 400,000 cases occur annually in the USA [4]. These infections are associated with a fatality rate of approximately 35% [5] and also significant increases the hospital stay [6-8]. Catheter-related infection (CRI) also contributes to the inappropriate and excessive use of antimicrobial agents and may lead to the selection of antibiotic-resistant organisms.

Early detection and adequate treatment of causative pathogens within 24 hours of clinical suspicion of these infections (development of signs and symptoms) is critical for a favourable outcome, yet the majority of patients with suspected CRI yield negative diagnostic investigations, necessitating empiric, rather than optimal antimicrobial therapy [9]. For example, in a study of 631 intensive care unit (ICU) catheters, 207 (33%) were removed due to clinical signs of CRI, yet definitive diagnosis from matched catheter and blood cultures was only achieved in 27 (13%), and catheter tip colonisation in 114 (55%) of suspected cases [10].

The current laboratory techniques for diagnosis of CRI include qualitative culture of the catheter tips, semi-quantitative culture of the catheter tips, quantitative culture of catheter segments (including the techniques of sonication, vortex or luminal flushing before catheter culture), and catheter staining methods such as with acridine orange [11]. These quantitative methods may have higher sensitivity, but are more time-consuming and complicated than semi-quantitive methods [11]. The semi-quantitative method is still the most commonly used method in clinical microbiology laboratories around the world [12] and remains the recommended method for routine clinical microbiological analysis [13].

The semi-quantitative method has however been criticised as regards its accuracy and delay of up to 2-4 days to provide culture results, therefore potentially delaying or missing the best treatment opportunity for patients with serious infections. Finally, the culture method is of limited value for slow-growing or fastidious bacteria, and for unculturable or intracellular pathogens, which can cause endocarditis (e.g. some Viridans Streptococci). The sensitivity of the semi-quantitative method may also be reduced if the patient is receiving antibiotic treatment. There is thus a need for the development of additional diagnostic methods to supplement conventional culture diagnosis, and molecular techniques have potential to fulfil this important role.

Arterial catheters (ACs) provide continuous, real-time blood pressure monitoring, easy, and rapid blood specimen access and are the most heavily manipulated catheters in critically ill patients [14]. It has been recently reported that the risk of AC-related bloodstream infections is close to that seen with short term central venous catheters (CVCs). Additionally AC colonisation rates have been demonstrated in critically ill patients to approximate those of short term CVCs [15]. Thus although ACs have been traditionally thought to have a much lower risk of infection [6,16-18] than short-term CVCs, this is no longer the case and current thinking suggests that they must be regarded with the CVC as a source of sepsis in critically ill patients [19].

The primary aim of this study was to assess the bacterial community on short term ACs in critically ill patients using culture-independent methods and compare these results with bacterial species diagnosed by the roll-plate semi quantitive method. The secondary aim of this study was to compare the bacterial community on 'colonised' and 'uncolonised' ACs. This study is the first comprehensive examination of bacterial communities on the surface of short-term ACs in critically ill patients.

## Methods

### Hospital setting and study population

The study setting was the ICU of the Royal Brisbane and Women's Hospital (RBWH), Queensland, Australia. This is a university-affiliated, mixed medical and surgical unit managing all forms of critically ill adult patients, except cardiac surgery and solid organ transplant patients. The unit is the sole referral centre for the management of severe burns trauma for the state of Queensland. During the study period (18 months), the ICU comprised 36 beds with admissions on average 2,000/annum. The mean (SD) patient Acute Physiology and Chronic Health Evaluation (APACHE) II score was of 16 ± 8.3 over this time period.

Patient management was not impinged upon by the study. Intravascular catheter management including insertion and removal was at the discretion of the treating clinician. All catheters were managed using a standardised protocol. All AC (Leader Cath, Vygon, Ecouen, France) were inserted by experienced ICU medical staff using a Seldinger approach. Aseptic precautions for all device insertion included use of a full sized drape, mask, cap, gown and sterile gloves. Chlorhexidine 2% was used for skin antisepsis. Ultrasound guided placement was used where required. There was no imposed limitation on dwell time, and resite of ACs always occurred at a new site. Dressings and administration sets were maintained by dedicated ICU nurses (1:1 nurse patient ratio) using unit protocols and in accordance with best evidence practice. All ACs were removed on suspicion of CRI by clinicians independent of the study, using the following criteria: intravascular device in situ; 2 or more SIRS criteria (Temperature >38.5°C or <36.0°C, Heart Rate >90 bpm, Respiratory Rate >20 bpm or PaCO2 <32 mmHg or requirement for mechanical ventilation, White Blood Cells >12 000 cells/mm^3 ^or <4000 cells/mm^3 ^or presence of >10% immature neutrophils); and no other source of the sepsis evident. All catheter tips were handled under aseptic conditions and immediately, transported to the laboratory for analysis, where they were cultured by the semi-quantitative method [12]. The cultivation and identification were performed by trained microbiologists in Microbiology Pathology Queensland-Central Laboratory, Australia.

Ninety three short-term ACs from four access sites (65 radial, 15 femoral, 7 brachial and 5 dorsalis pedis), with a mean catheter *in situ *time of 6.0 days, from 82 patients with a mean age of 51.0 years old and APACHE II score of 21.0, were studied. The mean ICU stay was 18.6 days with hospital survival of 86%. The arterial catheter related colonisation rates were 15.0/1000 device days and catheter related bloodstream infections rates were 3.8/1000 device days. These rates reflect the selection of the cohort as those suspected clinically of catheter related infection. There were no significant associations observed between antibiotic usage and AC colonisation or bloodstream infections (p = 0.126).

From this original cohort, 5 'colonised' and 5'uncolonised' ACs were randomly selected for further study (Table 1). The 5 colonised ACs comprised 2 mixed coagulase-negative *Staphylococci*, 2 *S. epidermidis *and 1 *P. aeruginosa*. No bacterial species were recovered from the uncolonised catheters using the semi-quantitive method.

**Table 1:** Comparison of the species richness, evenness, diversity of the 16S rRNA gene clones from two groups of ACs.

AC group	Catheter	Maki result	No. of	No. of	Richness indices		Evenness	Diversity index	
(based on	numbers		clones	OTUs			index		
Maki's results)				≥97%	Chao	ACE		Shannon	Simpson
Uncolonised ACs	1	No-growth	31	18					
						
	11	No-growth	24	19					
						
	16	No-growth	27	15					
					48	55	0.88	3.31	0.05
	17	No-growth	48	20					
						
	19	No-growth	48	20					
						
	Total		178	44					

Colonised ACs	3	mixed coagulase-negative *Staphylococci*	30	19					
						
	7	*S. epidermidis *>100 cfu	47	22					
						
	12	mixed coagulase-negative *Staphylococci*	90	39	61	69	0.81	3.20	0.07
						
	13	*S. epidermidis *>100 cfu	24	16					
						
	14	*P. aeruginosa *>100 cfu	48	19					
						
	Total		239	51					

Ethics approval for this study was granted by the Royal Brisbane and Women's Hospital Human Ethics Board (Protocol 2008/059) and Griffith University Human Ethics Board.

### Semi-quantitative method

The removal ACs were examined using the semi-quantitative method [12]. This method is based on rolling a segment, usually the tip, of the removed catheter back and forth on 5% sheep blood agar plates (Oxoid, Australia) after removal. The plates were incubated at 35°C under aerobic conditions for 2-4 days. Microorganisms were then isolated and identified according to standard hospital protocol. Semi-quantitative tip culture was considered colonised if the plate grew ≥15 colony forming unit (cfu). If <15 cfu were grown, the catheter tip was considered to be uncolonised.

### Detailed molecular methods

#### DNA extraction and PCR amplification

Catheter tips were suspended in 200 μl of lysis buffer, which contained 20 mg/ml lysozyme, 20 mM Tris-HCl (pH 8.0), 2 mM EDTA, 1.2% Triton, and Proteinase K at 37°C overnight. After that, catheter tips were taken out and bacterial DNA was extracted using the QIAamp DNA mini kit (Qiagen, Australia). For each catheter, a control (unused) AC was taken from the original packaging and rolled back and forth on blood agar plates, with bacterial DNA extracted as above. Sixteen S rRNA genes were amplified from purified genomic DNA using the primers 8F and 1490R [20]. For each 25 μl reaction, conditions were as follows: 3 μl of DNA template (concentration ranged from neat to 1:10^3^), 2.5 μl of 10 × reaction buffer containing 20 mM MgCl_2_, 2 μl of 25 mM dNTPs, 1 μl of each primer (10 μM), 0.1 U of *Taq *DNA polymerase (Qiagen, Australia), 5 μl of 5 × BSA and 10.4 μl of sterile deionised water (sdH_2_O). Each PCR run contained a negative control (sdH_2_O instead of template DNA) and a positive control (*E. coli *instead of template DNA). For each DNA sample, three replicate PCRs were performed. Thermocycling was as follows: initial denaturation at 95°C for 5 min, followed by 30 cycles of a 1-min denaturation, 1-min annealing at 55°C and 2-min elongation at 72°C, all followed by a final extension of 10 min at 72°C.

#### Cloning and sequencing of 16S rDNA PCR products

After purification using the Qiaquick PCR Purification kit (Qiagen, Australia), the PCR amplified 16S rRNA gene fragment were ligated into TOPO TA vector Cloning^® ^system (Invitrogen, Ausralia) according to the manufacturer's instructions. Two microliters of the ligation mixture was transferred to 1.5 ml sterile tube which was with competent *Escherichia coli *TOP10 cells provided by the manufacturer. The mixture was chilled on ice for 20 min before heat shocking for 45 seconds at 42°C. The cells were suspended with SOC medium and incubated with shaking at 37°C for one hour. The transformed cells were then plated onto Luria-Bertani (Promega, Australia) agar plates supplemented with kanamycin (Sigma, Australia) and incubated at 37°C overnight. Ninety six of the resulting bacterial colonies per ligation were picked and grown overnight at 37°C on LB agar plates containing kanamycin. Plasmid DNA was released from bacterial cells by boiling and one microliter was used as the template in PCR with an M13 forward and reverse primers to determine the correct sizes of inserts. The presence and size of inserts was determined by electrophoresing the PCR products on a 1% agarose gel. Subsequently positive PCR products were purified, lyophilized and sent to Macrogen Inc. (Seoul, South Korea) for sequencing using ABI PRISM^® ^BigDye™ and M13F vector-specific primer.

#### Alignment and phylogenetic analysis

The 16S rRNA gene clones of the arterial catheters were divided into two groups, i.e., uncolonised ACs and colonised ACs. The 16S rRNA gene sequences obtained were manually proofread, corrected and edited to start and end with the corresponding primer nucleotide (using reverse complement transform if necessary) using BioEdit [21]. Sequences with incorrect inserts or with ambiguous bases were excluded from further sequence analyses. Vector sequences detected by cross match were trimmed off. Trimmed, assembled sequences were then aligned to a core set of sequences using the NAST alignment tool on the Greengens website (http://greengenes.lbl.gov/cgi-bin/nph-index.cgi). All 16S rRNA gene sequences were screened for potential chimeras using BELLEROPHON which was also available on the Greengens website [22] and sequences flagged as potential chimeras were discarded from further analysis. Sequences were compared to the NCBI GenBank database using the BLAST program. All examined 16S rRNA gene clone sequences and their most similar GenBank sequences which were not available in the Greengenes database at the time of analysis were identified from BLAST searches of sequences retrieved in this study and were then imported into the ARB software package (http://www.arb-home.de) [23].

#### OTU determination and diversity estimation

The Olsen corrected distance matrix was exported from the ARB program and all sequences were grouped into operational taxonomic unit (OTUs) by the furthest-neighbour algorithm Distance-based Operational Taxonomic Unit and Richness (DOTUR). DOTUR assigned sequences accurately to OTUs based on sequence data using values that are less than the cut off level [24]. A cluster with less than 3% substitutions in the phylogenetic tree was usually matched with the same species or relatives in GenBank as confirmed by the RDP Classifier results. In this study, a similar cut off of 97% was defined as an OTU. This same cut off was used for diversity indices and richness estimates that were calculated by DOTUR. In this study, the Shannon and Simpson diversity indices, and Chao and ACE richness estimates were calculated by DOTUR to estimate microbial diversity and richness. Bacterial species evenness was also calculated [25]. The Chao richness estimator curves were continuously calculated during the sequencing phase. When the estimator curve reaches a plateau, the sequencing effort was considered to be sufficient to provide an unbiased estimate of OTU richness, as proposed by Kemp & Aller [26]. Rarefaction curve was generated by plotting the number of OTUs observed against number of sasequences sampled. The *P *value generated from two tailed *t*-test was used to determine significance of difference between different parameters.

### Nucleotide sequence accession numbers

The partial 16S rRNA gene sequences were deposited in the GenBank database and assigned accession numbers GQ476157-GQ476573.

## Results

### Composition of the 16S rRNA gene clone library

Bacterial DNA was extracted from all ten ACs, regardless of whether they were 'colonised' or 'uncolonised' as defined by the semi-quantitative roll-plate method. These DNA samples were successfully amplified and used for constructing 16S rRNA gene clone libraries. No bacterial DNA was detected from negative control ACs which proves bacterial presentation on ACs.

In the 16S rRNA gene clone library construction, 1,848 white colonies were identified including 926 from colonised ACs and 922 from uncolonised ACs. From these colonies, 980 (98 from each of the 10 ACs) were randomly selected, which accounted for 53.0% of the total clones. Among the clones, 430 clones were sequenced in total, obtaining 417 clone partial sequences. The lengths of the sequences for genetic comparison ranged between 771-867 bp, with an average for all the sequences of 808 bp. Most of the sequences matched a GenBank species or clone with an identity equal to or greater than 95% (396 out of 417). Chimera checks showed that all sequences were unlikely to be chimeric.

### Phylogenetic profiles and taxonomic distribution of the 16S rRNA gene clones among the ACs

All 417 sequences clustered into six groups (phyla or classes) according to the taxonomic classification of the NCBI database. These bacterial groups were *Firmicutes, Alphaproteobacteria, Betaproteobacteria, Gammaproteobacteria, Unclassified_Proteobacteria *and Unclassified Bacteria. The single most dominant division was *Gammaproteobacteria *(75.0%), which included *Xanthomonadales*-subdivision (45.9%), *Enterobacteriales*-subdivision (24.5%), and *Pseudomonadales*-subdivision (4.6%), followed by *Betaproteobacteria *(12%) which were all within *Burkholderiales*-subdivision, *Alphaproteobacteria *(8%), *Firmicutes *(4%) including *Staphylococcaceae*-subdivision (1.5%) and *Streptococcaceae*-subdivision (2.5%), *Unclassified proteobacteria *(0.5%) and Unclassified Bacteria (0.5%).

**Figure 1:**
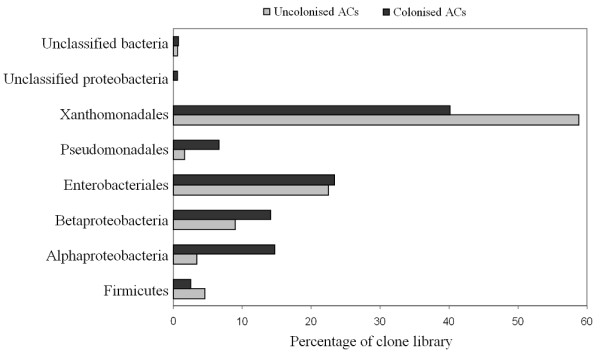
**Division level distribution of 16S rRNA gene clone sequences in uncolonised and colonised ACs**.

There were no significant differences between the uncolonised and colonised ACs in terms of the distribution of the taxonomic groups (Figure 1). *Firmicutes *accounted for approximate 4.50% and 2.53% of clone libraries from uncolonised and colonised ACs respectively. *Alphaproteobacteria *accounted for 12% in colonised ACs which was four times more than in uncolonised ACs. Similar trends were seen in *Pseudomonadales *which accounted for 6.6% in colonised ACs and only 1.69% in uncolonised ACs. Colonised ACs contained more *Betaproteobacteria*/*Burkholderiales *(14.07%) than uncolonised ACs (8.99%). Similar proportions of *Enterobacteriales*, *Xanthomonadales *and unclassified bacteria were observed in both groups. The difference between the overall distributions of the taxonomic groups in colonised and uncolonised ACs was not statistically significant (p = 0.976).

### OTU distribution among colonised and uncolonised ACs

**Figure 2:**
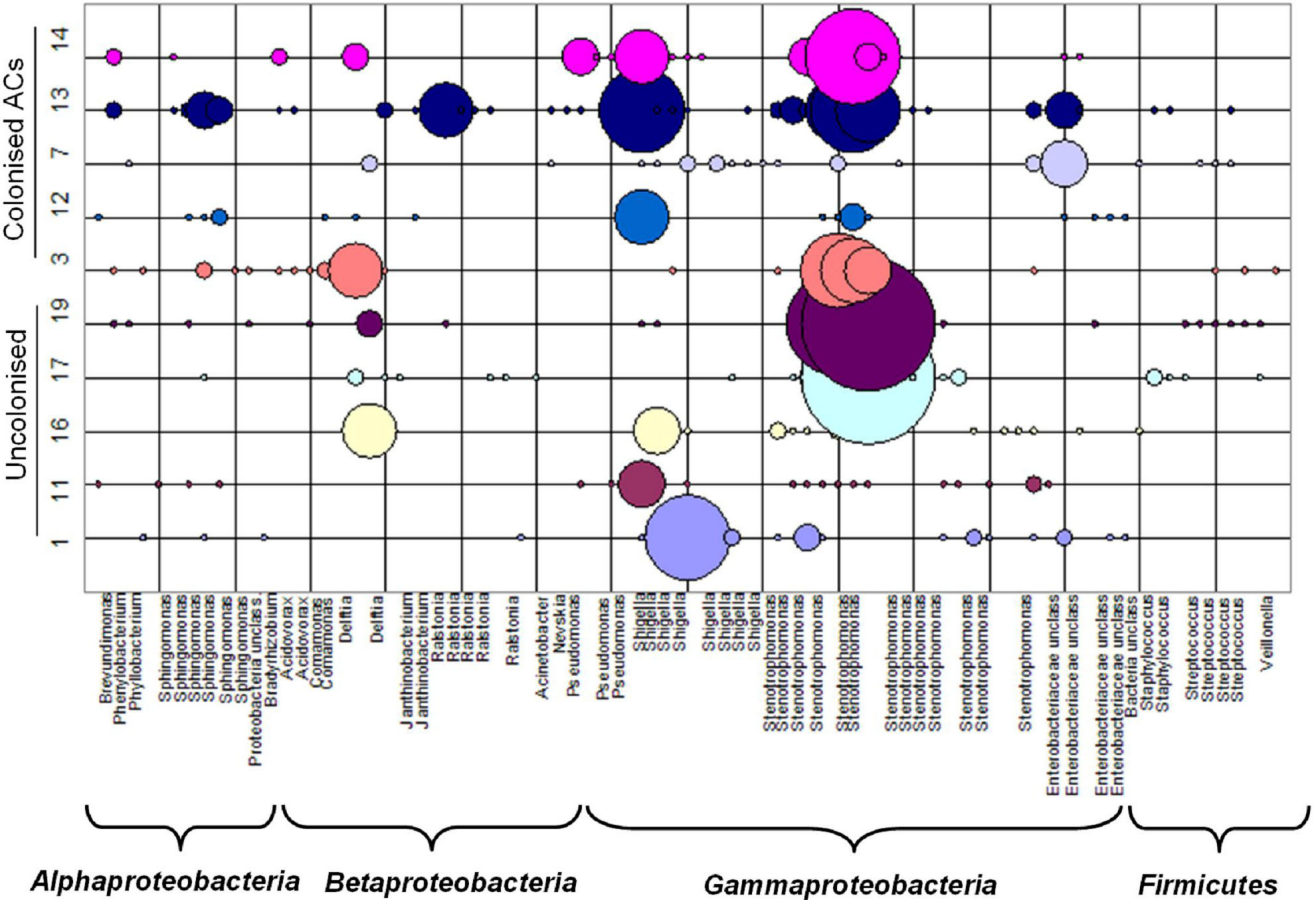
**Diversity of OTUs and their abundances in 16S rRNA gene clone libraries**. The taxonomic identity of each OTU was identified by phylogenetic analyses of the partial 16S rRNA gene sequences after separating them into the major bacterial phyla. A total of 79 OTUs were shown but not all the species names were labelled.

All of 417 sequences were grouped into OTUs based on their genetic distance in a neighbour-joining tree with the DOTUR program. Using the furthest-neighbour method of calculation and a similarity threshold of 97%, DOTUR assigned the 417 sequences into 79 OTUs. There is an average of 20 OTUs from each ACs including uncolonised and colonised devices. Approximately one quarter of the OTUs (21) were composed of a single sequence. However, three OTUs contained 30 or more sequences. The majority of OTUs and sequences belong to the division *Proteobacteria *with 86.1% and 95.9%, respectively for colonised and uncolonised ACs. The largest three OTUs, a member of the division *Gammaproteobacteria *and family *Xanthomonadaceae*, contained 191 sequences (45.8%). Other common *Proteobacteria *OTUs indentified included *Enterobacteriaceae, Pseudomonadaceae, Sphingomonadaceae, Comamonadaceae, Burkholderiaceae, Oxalobacteraceae, Caulobacteraceae, Phyllobacteriaceae*, and *Bradyrhizobiaceae *(Figure 2). OTUs and sequences were also identified from the division *Firmicutes *(11.4% and 4%, between colonised and uncolonised ACs respectively) including species of the family *Veillonellaceae, Staphylococcaceae*, and *Streptococcaceae*. We also identified two novel OTUs that were < 93% similar to any sequences in GenBank. These two OTUs were 92% and 91% similar to unknown clones from environmental samples. Overall there were 51 OTUs for colonised ACs and 44 OTUs uncolonised ACs. There were 33 and 27 single- and double-sequence OTUs for colonised and uncolonised ACs. Of the 79 OTUs identified in the two sets of samples, 40 (50.6%) were identified in both groups. However, these 40 OTUs represent 339 of 417 sequences (81.5%) of the clones. There was no significant difference between the distribution of sequences generated from colonised and uncolonised ACs in OTUs (p = 0.316).

**Figure 3:**
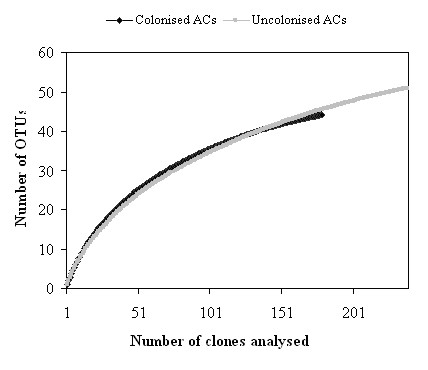
**Rarefaction analysis of 16S rRNA gene sequences**. All sequences were obtained from uncolonised and colonised ACs clone libraries using an OTU threshold of 97% identity.

The total species evenness (E) for the colonised and uncolonised ACs was 0.81 and 0.88 respectively. The total microbial richness for colonised and uncolonised ACs were calculated and estimated by Chao and ACE. Chao takes into account singletons and doubletons, while ACE uses OTUs having one to ten clones each. It was observed that OTU richness would increase with additional sequencing of clones. Both the Chao and ACE estimation for uncolonised ACs clone libraries were slightly lower than colonised ACs clone libraries (Table 1). As ACE and Chao are dependent of the amount of singletons, the discrepancies with the diversity indices are most probably due to different amounts of singletons in the clone libraries. From observed and estimated total richness for uncolonised and colonised ACs, we estimated that there was a minimum 5-10 more OTUs per group yet to be uncovered. However, it should be noted that no complex microbial community has even ever been sampled to completion. Rarefaction curve analyses (Figure 3) indicate that our sampling of clones is sufficient to give an overview of dominant microbial communities on the examined uncolonised and colonised ACs.

To estimate the relative diversity using 16S rRNA gene for colonised and uncolonised ACs, we calculated both Shannon and Simpson Diversity Indices, measures of ecosystem biodiversity. Each diversity index is associated with specific biases. The Shannon index places a greater weight on consistency of species abundance in OTUs, while the Simpson Index gives more weight to the abundance of OTUs. The Shannon's diversity index *H' *values for colonised and uncolonised ACs were 3.20 and 3.31 (Table 1). The Simpson diversity index values for colonised and uncolonised ACs were 0.93 and 0.95. Both indices suggest similar diversity profiles for both colonised and uncolonised ACs. The largest OTU from the colonised ACs contained 54 sequences and the OTU from the uncolonised ACs contained 26 sequences, which might explain the slightly lower diversity index values in colonised ACs. While these results suggested that the diversity indices in uncolonised ACs was slightly higher than colonised ACs, there was no significant difference between the two groups (p = 0.986).

## Discussion

Culture-independent methods have been successfully and widely used to reveal the microbial community in environmental and human samples [27-29]. Among these methods, the 16S rRNA gene clone screening approach provides a direct method for investigating bacterial diversity [27-29]. This study is the first attempt to use 16S rRNA gene clone screening approach to assess the bacterial community on surfaces of ACs taken from critically ill ICU patients with suspected catheter related blood-stream infections.

The results revealed a remarkable diversity of bacteria on ACs. It is generally believed that coagulase-negative staphycocci (such as *Staphylococcus epidermidis*) and *Staphylococcus aureus*, a subvision of phylum *Firmicutes *are the most dominant bacteria found in ACs although the infection rates might vary with different catheter insertion sites [30,31]. However, in this study the majority of sequences on ACs were from the division *Gammaproteobacteria*. The single most dominant subdivision was *Xanthomonadales *(*Stenotrophomonas maltophilia*).

A large number of bacterial clones in the libraries were from *Enterobacteriales*, *Pseudomonadales *and *Burkholderiales *which all contain pathogenetic species. Many of these bacteria are difficult to cultivate. Many of the examined clones were also closely related to known pathogens or opportunistic pathogens, but they were not identified by the semi-quantitative method. These sequences are the closest neighbours of *Staphylococcus epidermidis*, *Staphylococcus capitis*, *Streptococcus pyogenes*, *Streptococcus agalactiae*, *Stenotrophomonas maltophilia, Delftia acidovorans, Escherichia coli*, *Shigella flexneri*, *Comamonas testosteroni*, and *Brevundimonas diminuta. *Impressively, over 45% of clones examined in this study were *Stenotrophomonas maltophilia. *Over the last decade, *Stenotrophomonas maltophilia *has been documented as an important agent of nosocomial infection, including bloodstream infection, and has been associated with high mortality (26.7%) [32,33]. It was the third most frequent non-fermentative Gram-negative bacterium reported in the SENTRY Antimicrobial Surveillance Program between 1997 and 2001 [32]. Several reports on catheter-related bloodstream infections caused by *Stenotrophomonas maltophilia *exist [32-34]. *Stenotrophomonas *is increasingly recognised as a very important pathogen in the critically ill patient. In particular, it may become problematic in long stay patients who have been exposed to broad spectrum antibiotics. In this regard our result describing the abundance of this organism on ACs may have additional importance. In our ICUs this pathogen is not infrequently seen in this context, and treatment may be difficult due to resistance. *Shigella *species were also identified from both colonised and uncolonised ACs in this study. For a long time, it was believed that *Shigella *species were confined to the bowel and cause Shigellosis. However, several reports have now appeared in the literature of *Shigella *bacteraemia [35,36]. *Shigella *bacteraemia is still very rare and the mechanism of bacteraemia by *Shigella *species remains unclear [37]. Shigella was not however reported as a cause of bacteraemia arising from ACs. *Delftia acidovorans*, a bacterium known to be resistant to a class of drugs commonly used to treat systemic gram-negative infections (aminoglycosides) [38,39], was also identified in this study. Timely identification at species level is necessary to determine the most appropriate antibiotic therapy [38].

The results of the culture-dependent (semi-quantitive) and culture-independent (molecular) methods appear incongruent. Whereas semi-quantitive method reported the most frequently isolated bacteria from intravascular catheters as coagulase-negative staphylococci and *staphylococcus aureus *[16,40], our molecular data analysis from 16S rRNA gene clone sequences presented *Stenotrophomonas maltophilia *as the predominant bacteria. There are several reports of discrepancies between culture-dependent and culture-independent approaches for bacterial community studies [29,41,42]. Culture dependent methods bias bacteria who favour the growth media and grow fast under standard laboratory conditions. In addition, some bacterial species may compete with others for nutrients or they may even inhibit other bacteria from growing [20,41,43]. Unlike the semi-quantitive method, which only examines bacteria on outer surfaces of catheters, the molecular method used here enables assessing bacteria on both inner and outer surfaces of catheters. Together these factors might help explain variations of the bacterial community examined by these two methods. Compared to culture-dependent methods, culture-independent methods provide more comprehensive information on the bacterial community. The knowledge gained from this study may be a beginning step in improved understanding of pathogenesis and infection risks for critically ill patients with intravascular catheters. Replication of this study in other settings, as well as exploring the relationship between type and timing of commencement for antibiotic therapy, and diagnostic results, are important areas for future research.

## Conclusions

This study of critically ill patients with suspected CRI, has demonstrated that both colonised and uncolonised ACs examined by molecular method have an average of 20 OTUs per catheter, most of which are not isolated by the semi-quantitative method. Overall there were 79 OTUs in the two sets of samples which comprised 51 OTUs for colonised ACs and 44 OTUs uncolonised ACs. Of the 79 OTUs identified in the two sets of samples, 40 were identified in both groups. Statistically there was no significant difference in bacterial composition between uncolonised and colonised ACs, as confirmed by the results of *t*-test of taxonomic group distribution, the OTU distribution, and diversity indices. Taken together, this study suggests that in vascular devices removed for suspicion of CRI and analysed using semi-quantitative method, a negative culture result may not be indicative of non infective catheters. Moreover, these culture negative catheters may at times be a significant source of sepsis in critically ill patients. Whilst the clinical significance of these findings requires further study before any such conclusions may be drawn, the results suggest a need for the development of new methods that more accurately determine the presence of pathogens on intravascular devices.

## Authors' contributions

LZ performed all molecular work, data analysis and drafted the paper. JG collected the clinical samples and provided the clinical data. LZ, CR, KS, DM and JG participated in the design and coordination of the study and editing of the manuscript. BP participated in the design of the study. All authors read and approved the final manuscript.

## References

[B1] Edgeworth J Intravascular catheter infections J Hosp Infect 2009 73 4 323 330 10.1016/j.jhin.2009.05.008 19699555

[B2] Bouza E Alvarado N Alcala L Perez MJ Rincon C Munoz P A randomized and prospective study of 3 procedures for the diagnosis of catheter-related bloodstream infection without catheter withdrawal Clin Infect Dis 2007 44 6 820 826 10.1086/511865 17304454

[B3] Australian Infection Control Association National surveillance of healthcare associated infection in Australia: a report to the Commonwealth Department of Health and Aged Care 2001 1 225

[B4] Shukrallah B Hanna H Hachem R Ghannam D Chatzinikolaou I Raad I Correlation between early clinical response after catheter removal and diagnosis of catheter-related bloodstream infection Diagnostic Microbiology and Infectious Disease 2007 58 4 453 457 10.1016/j.diagmicrobio.2007.03.012 17509805

[B5] Crump JA Collignon PJ Intravascular catheter-associated infections Eur J Clin Microbiol Infect Dis 2000 19 1 1 8 10.1007/s100960050001 10706172

[B6] Bouza E Intravascular catheter-related infections: a growing problem, the search for better solutions Clin Microbiol Infect 2002 8 5 255 255 10.1046/j.1469-0691.2002.00479.x 12047401

[B7] Bouza E Burillo A Munoz P Catheter-related infections: diagnosis and intravascular treatment Clin Microbiol Infect 2002 8 5 265 274 10.1046/j.1469-0691.2002.00385.x 12047403

[B8] Mermel LA Farr BM Sherertz RJ Raad II O'Grady N Harris JS Craven DE Guidelines for the management of intravascular catheter-related infections Infect Control Hosp Epidemiol 2001 22 4 222 242 10.1086/501893 11379714

[B9] Timsit JF Diagnosis and prevention of catheter-related infections Current Opinion in Critical Care 2007 13 5 563 571 10.1097/MCC.0b013e3282efa03f 17762237

[B10] Valles J Fernandez I Alcaraz D Chacon E Cazorla A Canals M Mariscal D Fontanals D Moron A Prospective randomized trial of 3 antiseptic solutions for prevention of catheter colonization in an intensive care unit for adult patients Infect Control Hosp Epidemiol 2008 29 9 847 853 10.1086/590259 18665819

[B11] Linares J Dominguez MA Martin R Current laboratory techniques in the diagnosis of catheter-related infections Nutrition 1997 13 4 S10 S14 10.1016/S0899-9007(97)00216-5 9178304

[B12] Maki DG Weise CE Sarafin HW A semiquantitative culture method for identifying intravenous catheter-related infections N Engl J Med 1977 296 1305 1309 10.1056/NEJM197706092962301 323710

[B13] Mermel LA Allon M Bouza E Craven DE Flynn P O'Grady NP Raad II Rijnders BJA Sherertz RJ Warren DK Clinical Practice Guidelines for the Diagnosis and Management of Intravascular Catheter-Related Infection: 2009 Update by the Infectious Diseases Society of America Clin Infect Dis 2009 49 1 1 45 10.1086/599376 19489710 PMC4039170

[B14] Maki DG Kluger DM Crnich CJ The risk of bloodstream infection in adults with different intravascular devices: A systematic review of 200 published prospective studies Mayo Clinic Proceedings 2006 81 9 1159 1171 10.4065/81.9.1159 16970212

[B15] Koh DBC Gowardman JR Rickard CM Robertson IK Brown A Prospective study of peripheral arterial catheter infection and comparison with concurrently sited central venous catheters Crit Care Med 2008 36 2 397 402 10.1097/CCM.0b013e318161f74b 18216598

[B16] Raad II Hanna HA Intravascular catheter-related infections - New horizons and recent advances Arch Intern Med 2002 162 8 871 878 10.1001/archinte.162.8.871 11966337

[B17] Lorente L Villegas J Martin MM Jimenez A Mora ML Catheter-related infection in critically ill patients Intensive Care Medicine 2004 30 8 1681 1684 10.1007/s00134-004-2332-3 15160239

[B18] Lorente L Santacreu R Martin MM Jimenez A Mora ML Arterial catheter-related infection of 2,949 catheters Critical Care 2006 10 3 10.1186/cc4930 PMC1550952 16723035

[B19] Gowardman JR Lipman J Rickard CM Assessment of peripheral arterial catheters as a source of sepsis in the critically ill: a narrative review J Hosp Infect 2010 75 1 12 18 10.1016/j.jhin.2010.01.005 20303618

[B20] Zhang L Xu ZH Patel B Culture-dependent and culture-independent microbial investigation of pine litters and soil in subtropical Australia Journal of Soils and Sediments 2009 9 2 148 160 10.1007/s11368-009-0059-z

[B21] Hall-Stoodley L Stoodley P Biofilm formation and dispersal and the transmission of human pathogens Trends Microbiol 2005 13 7 300 301 10.1016/j.tim.2005.05.004 15639625

[B22] Huber T Faulkner G Hugenholtz P Bellerophon: a program to detect chimeric sequences in multiple sequence alignments Bioinformatics 2004 20 14 2317 2319 10.1093/bioinformatics/bth226 15073015

[B23] Ludwig W Strunk O Westram R Richter L Meier H Yadhukumar Buchner A Lai T Steppi S Jobb G ARB: a software environment for sequence data Nucleic Acids Research 2004 32 4 1363 1371 10.1093/nar/gkh293 14985472 PMC390282

[B24] Schloss PD Handelsman J Introducing DOTUR, a computer program for defining operational taxonomic units and estimating species richness Appl Environ Microbiol 2005 71 3 1501 1506 10.1128/AEM.71.3.1501-1506.2005 15746353 PMC1065144

[B25] Rani A Sharma A Rajagopal R Adak T Bhatnagar RK Bacterial diversity analysis of larvae and adult midgut microflora using culture-dependent and culture-independent methods in lab-reared and field-collected Anopheles stephensi-an Asian malarial vector BMC microbiology 2009 9 96 118 10.1186/1471-2180-9-96 19450290 PMC2698833

[B26] Kemp PF Aller JY Bacterial diversity in aquatic and other environments: what 16S rDNA libraries can tell us FEMS Microbiology Ecology 2004 47 2 161 177 10.1016/S0168-6496(03)00257-5 19712332

[B27] Grice EA Kong HH Renaud G Young AC Bouffard GG Blakesley RW Wolfsberg TG Turner ML Segre JA Sequencing NC A diversity profile of the human skin microbiota Genome Research 2008 18 7 1043 1050 10.1101/gr.075549.107 18502944 PMC2493393

[B28] Marchini L Campos MS Silva AM Paulino LC Nobrega FG Bacterial diversity in aphthous ulcers Oral Microbiology and Immunology 2007 22 4 225 231 10.1111/j.1399-302X.2006.00345.x 17600533

[B29] Paster BJ Boches SK Galvin JL Ericson RE Lau CN Levanos VA Sahasrabudhe A Dewhirst FE Bacterial diversity in human subgingival plaque Journal of Bacteriology 2001 183 12 3770 3783 10.1128/JB.183.12.3770-3783.2001 11371542 PMC95255

[B30] Lorente L Jimenez A Jimenez JJ Iribarren JL Martin MM Mora ML The catheter site influences in the micro-organism responsible of arterial catheter-related infection Intensive Care Medicine 2006 32 11 1919 1920 10.1007/s00134-006-0388-y 17019541

[B31] Lorente L Mora ML Jimenez A Microorganisms responsible for femoral catheter-related bloodstream infection Crit Care Med 2008 36 2 657 658 10.1097/CCM.0b013e318162b712 18216644

[B32] Friedman ND Korman TM Fairley CK Franklin JC Spelman DW Bacteraemia due to Stenotrophomonas maltophilia: An analysis of 45 episodes J Infect 2002 45 1 47 53 10.1053/jinf.2002.0978 12217732

[B33] Wang WS Liu CP Lee CM Huang FY Stenotrophomonas maltophilia bacteremia in adults: four years' experience in a medical center in northern Taiwan J Microbiol Immunol Infect 2005 37 359 365 15599468

[B34] Micozzi A Venditti M Monaco M Friedrich A Taglietti F Santilli S Martino P Bacteremia due to Stenotrophomonas maltophilia in patients with hematologic malignancies Clin Infect Dis 2000 31 3 705 711 10.1086/314043 11017819

[B35] Liu CY Huang YT Liao CH Chang SC Hsueh PR Rapidly Fatal Bacteremia Caused by Shigella sonnei without Preceding Gastrointestinal Symptoms in an Adult Patient with Lung Cancer Clin Infect Dis 2009 48 11 1635 1636 10.1086/598994 19416029

[B36] Davies NECG Karstaedt AS Shigella bacteraemia over a decade in Soweto, South Africa Transactions of the Royal Society of Tropical Medicine and Hygiene 2008 102 12 1269 1273 10.1016/j.trstmh.2008.04.037 18550134

[B37] Bello CS Al-Barki AA El-Awad ME Patel RV Shigella flexneri bacteremia in a child Saudi Medical Journal 2003 24 4 403 405 12754544

[B38] Oliver JW Stapenhorst D Warraich I Griswold JA Ochrobactrum anthropi and Delftia acidovorans to bacteremia in a patient with a gunshot wound Infect Dis Clin Practice 2005 13 78 81

[B39] Castagnola E Tasso L Conte R Nantron M Barretta A Giacchino R Central Venous Catheter-Related Infection Due to Comamonas-Acidovorans in a Child with Non-Hodgkins-Lymphoma Clin Infect Dis 1994 19 3 559 560 7811890 10.1093/clinids/19.3.559-a

[B40] Kamal GD Pfaller MA Rempe LE Jebson PJR Reduced intravascular infection by antibiotic bonding - reply J Am Med Assoc 1991 266 11 1514 1514 10.1001/jama.266.11.1514

[B41] Grice EA Kong HH Conlan S Deming CB Davis J Young AC Bouffard GG Blakesley RW Murray PR Green ED Topographical and temporal diversity of the human skin microbiome Science 2009 324 5931 1190 1192 10.1126/science.1171700 19478181 PMC2805064

[B42] Lee L Tin S Kelley ST Culture-independent analysis of bacterial diversity in a child-care facility BMC microbiology 2007 7 27 10.1186/1471-2180-7-27 17411442 PMC1853100

[B43] Mancini N Carletti S Ghidoli N Cichero P Burioni R Clementi M The Era of Molecular and Other Non-Culture-Based Methods in Diagnosis of Sepsis Clinical Microbiology Reviews 2010 23 1 235 251 10.1128/CMR.00043-09 20065332 PMC2806664

